# Efficacy and immunogenicity of insulin biosimilar compared to their reference products: a systematic review and meta-analysis

**DOI:** 10.1186/s12902-022-00944-5

**Published:** 2022-02-05

**Authors:** Li-Jou Yang, Ta-Wei Wu, Chao-Hsiun Tang, Tzu-Rong Peng

**Affiliations:** 1 Department of Pharmacy, Taipei Tzu Chi Hospital, Buddhist Tzu Chi Medical Foundation, #289, Jianguo Road, Xindian Dist, New Taipei City, 23142 Taiwan, Republic of China; 2 School of Health Care Administration, College of Management, Taipei Medical University, Taipei, Taiwan

**Keywords:** Biosimilar, Insulins, HbA1C, Fasting plasma glucose

## Abstract

**Background:**

To ascertain the efficacy, safety, and immunogenicity from existing evidence via conducting a meta-analysis of randomized controlled trials between biosimilar and originator insulins.

**Methods:**

The PubMed, Cochrane Library, EMBASE, and ClinicalTrails.gov were searched to identify head-to-head randomized controlled trials (RCTs) that directly compare the efficacy and safety of biosimilar insulin and its originator. Efficacy was assessed by change of HbA1C, fasting plasma glucose (laboratory or self-monitoring of blood glucose (SMBG)), and change all mean of 7 points- or 8 points- SMBG. Safety was assessed by change in proportion hypoglycemia and serious hypoglycemia. The occurrence of anti-insulin antibodies (AIAs) was also evaluated.

**Results:**

Fourteen RCTs with 6188 patients from different countries were included. Data were pooled using a random-effects model and were expressed as the mean difference (MD), odds ratio (OR), and 95% confidence interval (CI). In efficacy, Insulin biosimilar products showed similar in change of HbA1C at weeks 26 and 52, the MD were 0.03 (95% CI − 0.02 to 0.07, *p* = 0.28), and 0.05 (95% CI − 0.05 to 0.15, *p* = 0.36), respectively. The proportion of HbA1C less than 7% at endpoint, the OR were 1.04 (95% CI 0.89 to 1.20, *p* = 0.64). The change of fasting plasma glucose (laboratory or SMBG) mmol/L in 24–52 weeks and change all mean of 7 points−/8 points- SMBG mmol/L in 24–52 weeks, the MD were 0.02 (95% CI − 0.20 to 0.24, *p* = 0.87) and − 0.34 (95% CI − 1.35 to 0.67, *p* = 0.51), respectively. In occurrence of hypoglycemia (≥ 1 events) and severe hypoglycemia, the OR were 0.96 (95% CI 0.85 to 1.09, *p* = 0.52) and 1.06 (95% CI 0.85 to 1.31, *p* = 0.62). The AIA was 1.02 (95% CI 0.90 to 1.16, *p* = 0.76). Analysis stratified by type of diabetes and duration of insulin. There was no significant difference between the biosimilar and their reference group in a different type of diabetes and different duration of insulin.

**Conclusions:**

Insulin biosimilar showed comparable characteristics with the reference drug in terms of efficacy, safety, immunogenicity, through comprehensive and specific conventional meta-analysis.

**Supplementary Information:**

The online version contains supplementary material available at 10.1186/s12902-022-00944-5.

## Background

Diabetes mellitus (DM) is considered a complex and chronic disease [1]. In 2019, approximately 463 million adults (20–79 years) were living with diabetes; by 2045 this will rise to 700 million [2]. Due to incremental population in DM and influence all age group of adults. The management of patients living with type 1 and type 2 diabetics, and their complications, such as cardiovascular disease, is highly important and influences the disease mortality [3]. The full global costs of diabetes in adults will increase in costs as a share of global gross domestic product (GDP) from 1.8% in 2015 to a maximum of 2.2% in 2030. The global costs of diabetes and its consequences are large and will substantially increase [4]. Good glycemic control not only is the goal of diabetes treatment but also prevents cardiovascular complications and reduces mortality consequently [5, 6]. Patients living with type 1 diabetes must use insulin. However, if patients living with type 2 diabetes, medications include oral medications to control it, while others may also need insulin. Insulins biosimilar has expanded treatment options for diabetes and can potentially reduce medical costs, especially in low- and middle-income countries. Biosimilar has an important role in its economic benefit. The notable cost-effectiveness impact of a biosimilar is the potential for healthcare budgetary redistribution, decrease financial barriers and increase patient access to biological therapies [7, 8]. The benefit of this research will not only contribute to low-middle income countries but will also influence other high-income countries. Therefore, this study must determine whether the effect is consistent before confirming the cost-effectiveness. Although the results in clinical trials are consistent, when the number of samples increases, it may cause differences or differences in certain subgroups.

A health authority-approved biosimilar product is considered to have comprehensive evidence on its structure, biological activity and efficacy, safety, and immunogenicity profile to its reference product [9, 10]. Insulins are biological agents manufactured by complex processes, also costly and time-consuming to perform numbers phase 3 studies [11].

Since September 2014, the first insulin biosimilar has been approved by European Medicines Agency, there is a subsequent insulin biosimilar that has been approved by different Health Authority, and marketing worldwide [12]. Several insulins biosimilar has conducted Phase III studies for marketing authorization applications to show the treatment similarity between the biosimilar product and their reference product. However, there are still limited subjects to be included in the Clinical trial. The hypothesis of the meta-analysis might be no clinically significant difference between insulin biosimilar and innovator products in efficacy, safety, and immunogenicity. Hence, in this study, we aim to provide evidence of accumulated clinical trials for insulin biosimilar and clarify the efficacy, safety, and immunogenicity from existing evidence by performing a meta-analysis of randomized controlled trials between biosimilar and originator insulins.

## Methods

### Literature search strategy

We conducted a search on PubMed, Cochrane Library, Embase and ClinicalTrials.gov through 30th Nov 2021, limiting to human patients and publications in English. The following search terms were included in the search: insulin and biosimilar (Additional file 1: Appendix S1). All retrieved abstracts, studies, and citations were reviewed. The details of the search strategy for eligible studies are given in the flowchart provided by Preferred Reporting Items for Systematic Reviews and Meta-Analyses [13]. Two reviewers (L. J. Y. and T. R. P.) screened all titles and abstracts independently and evaluated relevant articles.

### Selection of relevant studies and criteria

This study was performed by Cochrane Collaboration guidelines [14]. We included trials that met the following criteria: 1) all studies included were phase III, head-to-head, non-inferiority, randomized control design, 2) comparison of any insulin biosimilar [non-originator product of investigated International nonproprietary name (INN)] and its reference product (originator of investigated INN) and report efficacy, safety, and immunogenicity outcome 3) inclusion of all diabetes mellitus types, and subcutaneous administration of insulin and 4) mentioned patient inclusion (including diagnoses of disease, age, insulin therapy, Body Mass Index) and exclusion criteria (experience severe hypoglycemia or hypoglycemia need further emergency room or hospitalization service for glucose control before inclusion), the outcome of efficacy, safety and immunogenicity, and treatment procedures for all groups.

### Data extraction

The following information was extracted: author, year of publication, study design, sample sizes, type of diabetes, duration of DM, clinical efficacy [change of HbA1C (%) from baseline, FPG (laboratory or SMBG) mmol/L, 7 points/8 points- SMBG mmol/L], and safety [hypoglycemia (≥1 events) %; severe hypoglycemia (d/N)], immunogenicity [anti-insulin antibody (%)].

### Risk of bias of included studies

Two reviewers (L. J. Y. and T. R. P.) independently assessed the quality of the included studies by using the revised risk-of-bias (version 2.0) method, according to the recommendation of the Cochrane Collaboration [15]. These cover the potential sources of bias including selection bias (random sequence generation and allocation concealment), performance bias (blinding of participants and personnel), detection bias (blinding of outcome assessment), attrition bias (incomplete outcome data), and reporting bias (selective reporting).

### Statistical analyses

Statistical analysis was performed according to the Cochrane Handbook for Statistical Review of Interventions (version 6.2) [14]. The statistical analyses were performed using RevMan software (Cochrane Review Manager Version 5.4, Oxford, UK) and Comprehensive Meta-Analysis software. Weighted mean differences (MD) and pooled odds ratios (ORs) were calculated by DerSimonian–Laird random-effects meta-analysis [16]. We assessed heterogeneity using a χ2 test with *p* < 0.10 considered statistically significant. Heterogeneity was considered low, moderate, or high for *I*^*2*^ values of < 25, 25–50, and > 50%, respectively. Results were considered statistically significant with a *p* value of < 0.05. We used funnel plot to assess the publication bias. The Egger’s and Begg’s tests were also used. A *p*-value of > 0.05 based on the results of Egger’s and Begg’s tests indicated the absence of publication bias.

## Results

### Study characteristics

A total of 196 records were screened and 89 full-text articles were assessed for eligibility. Fourteen articles were selected for qualitative review [17–30]. The trial selection procedure is shown in Fig. S1. The studies involved 6188 participants from more than 5 countries. All the studies reported the outcome of efficacy and safety and eleven of them also reported immunogenicity [19, 20, 22–30]. Types of insulin biosimilar were involved, 9 studies investigated the long-acting insulin (insulin glargine), including Basalog (Biocon Ltd., Bangalore, India), LY-IGlar (Lily, IN, USA), MYL-1510D (Mylan INC., IN, USA), MK-1293(Merk, NJ, USA), GP40061 (Geropharm, Russian); 4 studies investigated short-acting (insulin aspart, insulin lispro), such as SAR342434, SAR341401 (Sanofi, Paris, France), GP-Asp (Geropharm, Russia); 1 study investigated pre-mixed insulin biosimilar GP-Lis25 (Geropharm, Russia). The characteristics of the 14 studies are summarized in Table 1. The clinical outcomes of the trials are summarized in Table S1. There was a low risk of bias, except for blinding of participants and study personnel. Thirteen studies were open-label, and one was double-blind randomized control trials (Fig. S2).

**Table 1:** Characteristics of included studies

Study, Year	Trial name	Sample size (BSM/REF)	BSM vs REF	Race	Study/Trial design	Type of DM	Time point, wk	Duration of DM (y)	HbA1C, %	FPG, mmol/L	BMI, Kg/m2	ADA (+), %
**Long-acting insulin biosimilar**												
Verma, 2011 [17]	NA	107/108	Basalog vs Lantus	Indian	RCT/Non-inferiority	1	12	11	7.9	8.1	21.8	NA
Kaku, 2016 [18]	NA	131/129	FFP-112 vs Lantus	Japanese	RCT/Non-inferiority	1	24/52	NA	7.8	7.8	NA	NA
Blevins, 2015 [19]	ELEMENT 1	268/267	LY Iglar vs Lantus	White (74%), Asian (18%), American-Indian	RCT/Non-inferiority	1	24/52	17	7.8	8.3	25.5	0.7
Rosenstock, 2015 [20]	ELEMENT 2	376/380	LY Iglar vs Lantus	White (80%), Asian, Black	RCT/Non-inferiority	2	24	12	8.3	8.8	32	NA
Blevins, 2018 [21]	INSTRIDE 1	280/278	MYL-1501D vs Lantus	Europe USA	RCT/Non-inferiority	1	24/52	19	7.4	9.2	26.5	NA
Home, 2018 [22]	NA	245/263	MK-1293 vs Lantus	White (80%), Asian, Black	RCT/Non-inferiority	1	24/52	22	8	9.3	26.4	58
Hollander, 2018 [23]	NCT02059187	266/265	MK-1293 vs Lantus	White (63%), Black (12.5%), Multi-racial (15%), Asian	RCT/Non-inferiority	2	24	13	8.4	8.5	32.3	32
Blevins, 2019 [24]	INSTRIDE 2	277/283	MYL-1501D vs Lantus	North America, East Asia, Eastern Europe, and other regions	RCT/Non-inferiority	2	24	12	8.1	8.6	31.6	NA
Karonova, 2020 [25]	NCT04022993	90/90	GP40061 vs Lantus	caucasian	RCT/Non-inferiority	1	26	14	8.6	11	24.3	NA
**Short-acting insulin biosimilar**												
Garg, 2017 [26]	SORELLA 1, 2017	253/254	SAR232434 vs Humalog	NA	RCT/Non-inferiority	1	26/52	19	8	10	NA	48
Derwahl, 2018 [27]	SORELLA 2, 2017	253/254	SAR232434 vs Humalog	US, EU, RoW	RCT/Non-inferiority	2	26	17	8	NA	32.2	25
Garg, 2020 [28]	GEMELLI 1	301/296	SAR341402 vs NovoRapid	US, EU, RoW	RCT/Non-inferiority	1.2	26	19	8	NA	27.5	36
Karonova, 2021 [29]	NCT04079413	132/132	GP-Asp vs NovoRapid	European	RCT/Non-inferiority	1	26	13.5	8.6	11	24.8	8.3
**Pre-mixed insulin biosimilar**												
Mayorov, 2020 [30]	NCT04023344	105/105	GP-Lis25 vs Humalog Mix 25	Russia	RCT/Non-inferiority	2	26	10	9.4	11.8	31	NA

*ADA* (+)Anti-Drug-Antibody positive, *BMI* body mass index, *BSM* biosimilar, *BW* body weight, *DM* diabetes mellitus, *FPG* fasting plasma glucose, *IGlar* Lantus insulin glargine, *LY IGlar* LY2963016 insulin glargine, *Ly-Lis* Insulin lispro-Humalog, *MYL lGar* MYL-1501D (Mylan’s insulin glargine), *MK IGlar* MK-1293 insulin glargine, *NA* not available, *REF* reference, *SAR-Lis* SAR342434 insulin lispro, *SD* standard deviation

**Fig. 1:**
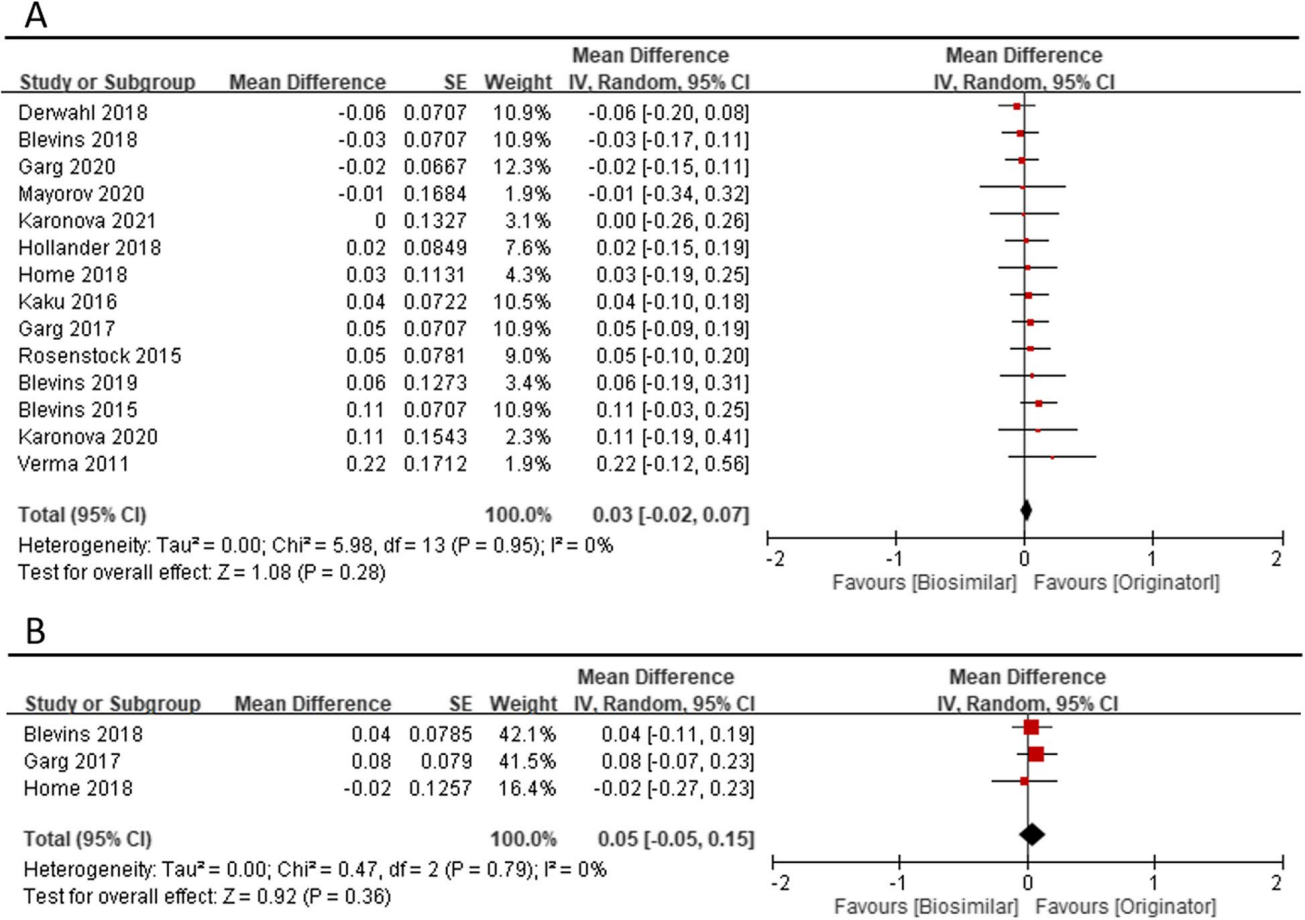
Change of HbA1C (%), (**A**) at 26 weeks (**B**) at 52 weeks

**Fig. 2:**
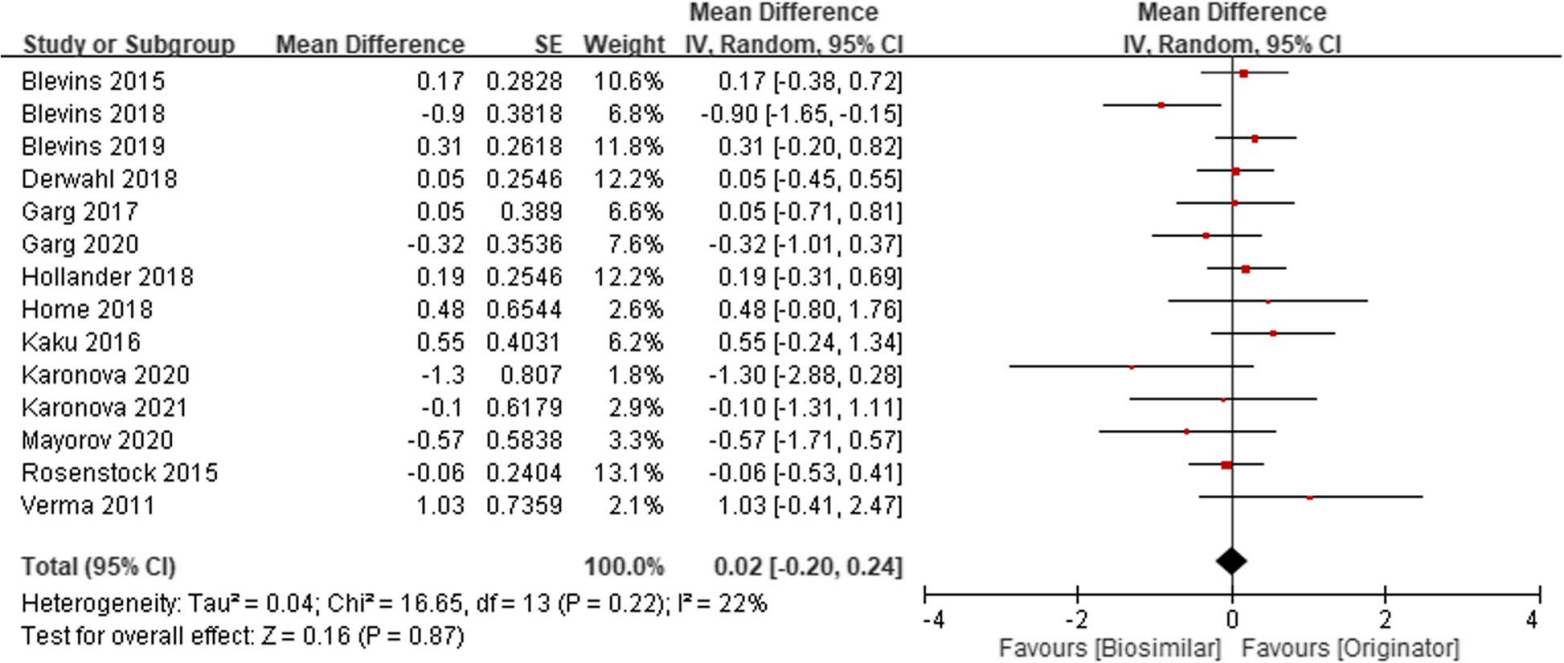
Change of fasting plasma glucose (laboratory or self-monitoring of blood glucose) (mmol/L) at 24–52 weeks

**Fig. 3:**
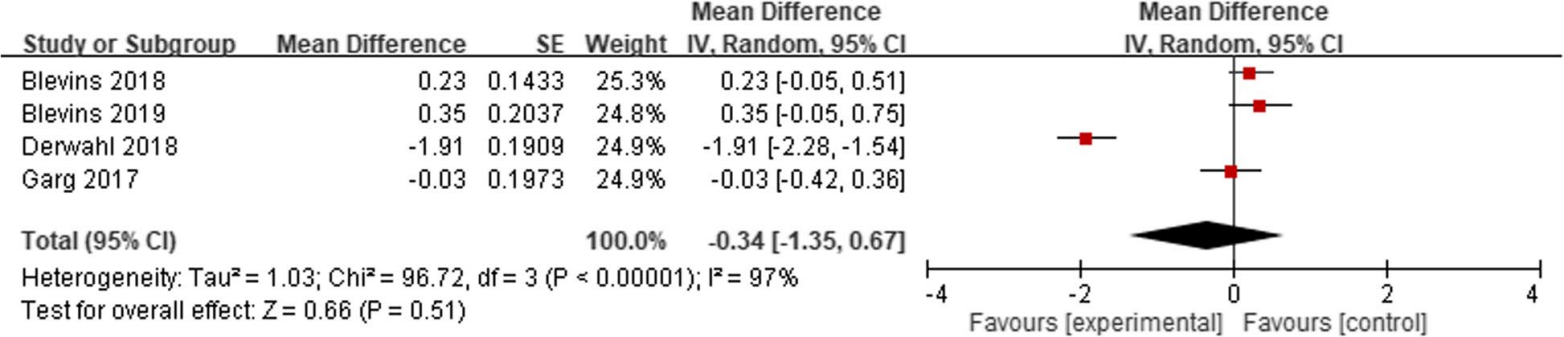
Change all mean of 7 points- or 8 points- self-monitoring of blood glucose (mmol/L) at 24–52 weeks

**Fig. 4:**
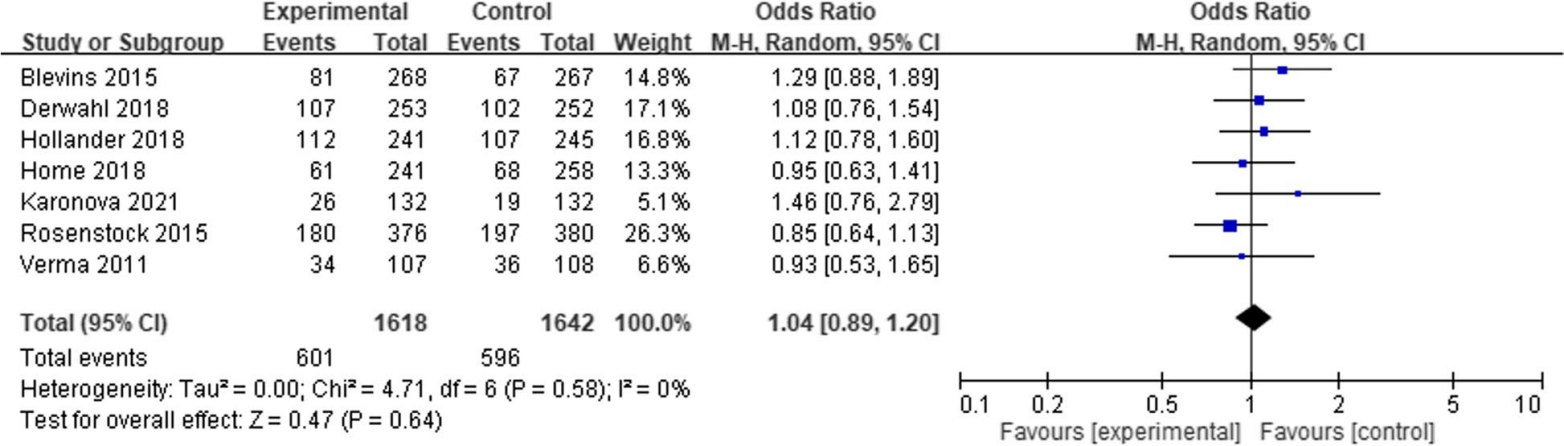
Achieving HbA1C < 7%

Meta-analyses of the clinical efficacy, blood sugar management in change HbA1C at 26 weeks (14 studies), change HbA1C at 52 weeks (3 studies) were performed according to follow-up period. The mean difference (MD) of change of HbA1C compared with baseline were 0.03 (95% CI − 0.02 to 0.07, *p* = 0.28), and 0.05 (95% CI − 0.05 to 0.15, *p* = 0.36), respectively (Fig. 1). The MD of HbA1C change from baseline in 26 weeks and 52 weeks shows clinical similarity. The pooled MD of fasting plasma glucose (laboratory or SMBG) mmol/L in 24–52 weeks among included 14 studies, were 0.02 (95% CI − 0.20 to 0.24, *p* = 0.87) (Fig. 2). The pooled MD of 7 points−/8 points- SMBG mmol/L in 24–52 weeks (4 studies) decreased from baseline but was not significantly different between the two groups. [− 0.34 (95% CI − 1.35 to 0.67, *p* = 0.51)] (Fig. 3). Seven studies also report proportion of HbA1C less than 7% at endpoint, the odds ratio were 1.04 (95% CI 0.89 to 1.20, *p* = 0.64) (Fig. 4).

**Fig. 5:**
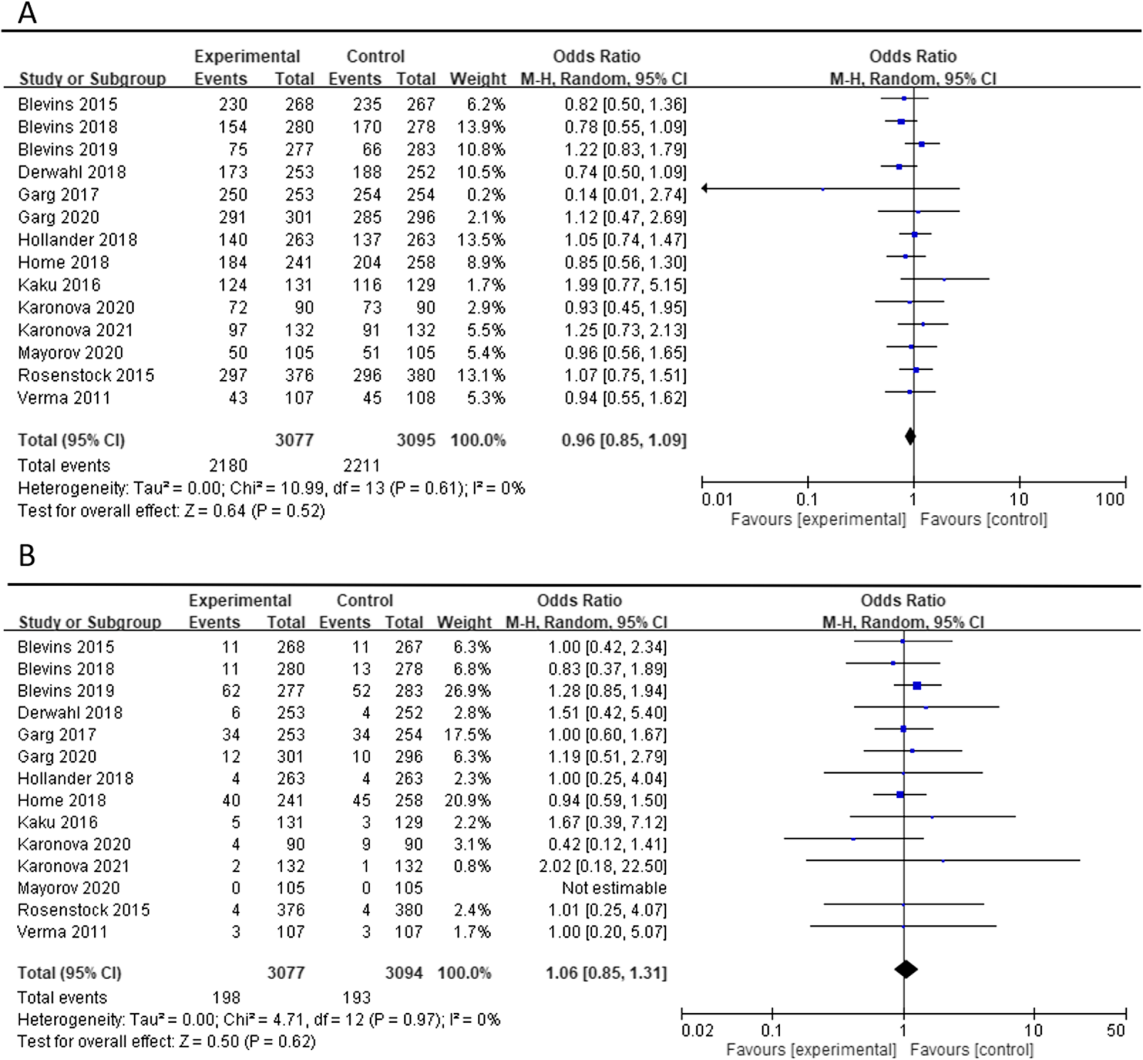
Hypoglycemia (**A**) Hypoglycemia ≥1 event (**B**) Severe hypoglycemia

**Fig. 6:**
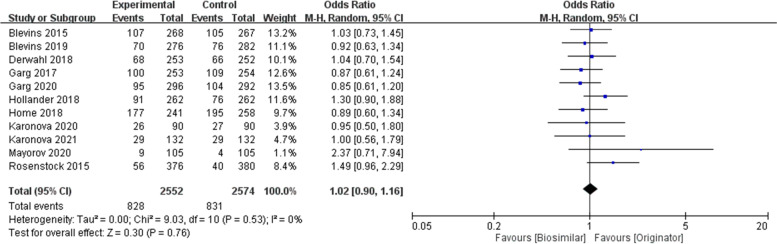
Anti-insulin antibody positivity (%)

The safety-related outcome in the hypoglycemia proportion (≥ 1 events) and severe hypoglycemia were assessed. The ORs of hypoglycemia proportion (≥ 1 events) among included 14 studies were 0.96 (95% CI 0.85 to 1.09, *p* = 0.52) and severe hypoglycemia among included 14 studies were 1.06 (95% CI 0.85 to 1.31, *p* = 0.62) (Fig. 5). There was no significant difference in safety-related outcomes. The percentage of AIA between the biosimilar and reference products showed no significant difference [1.02 (95% CI 0.90 to 1.16, *p* = 0.76)] (Fig. 6).

### Subgroup, and sensitivity analysis

**Fig. 7:**
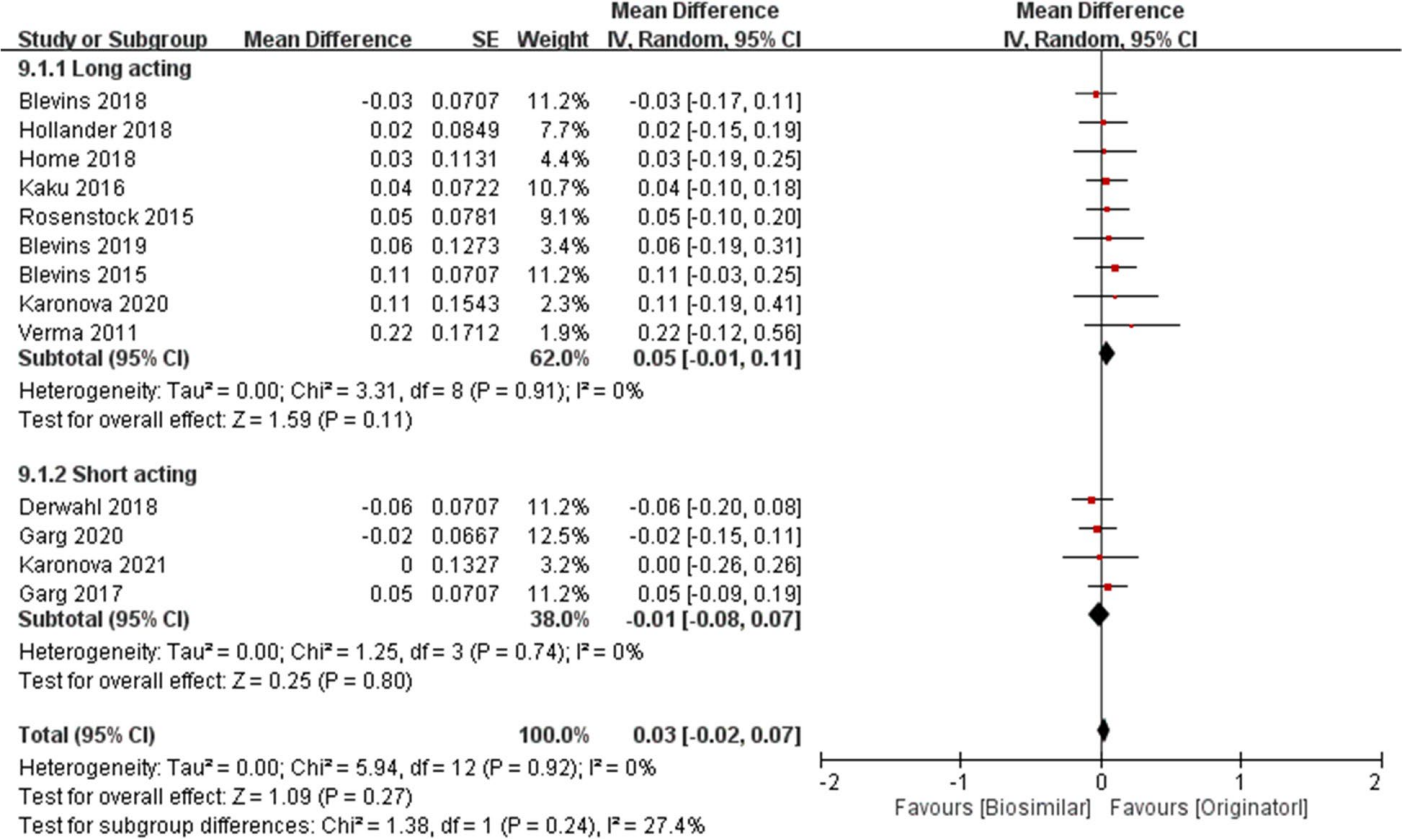
HbA1C at 26 weeks (%) in long-acting and short-acting insulins

**Fig. 8:**
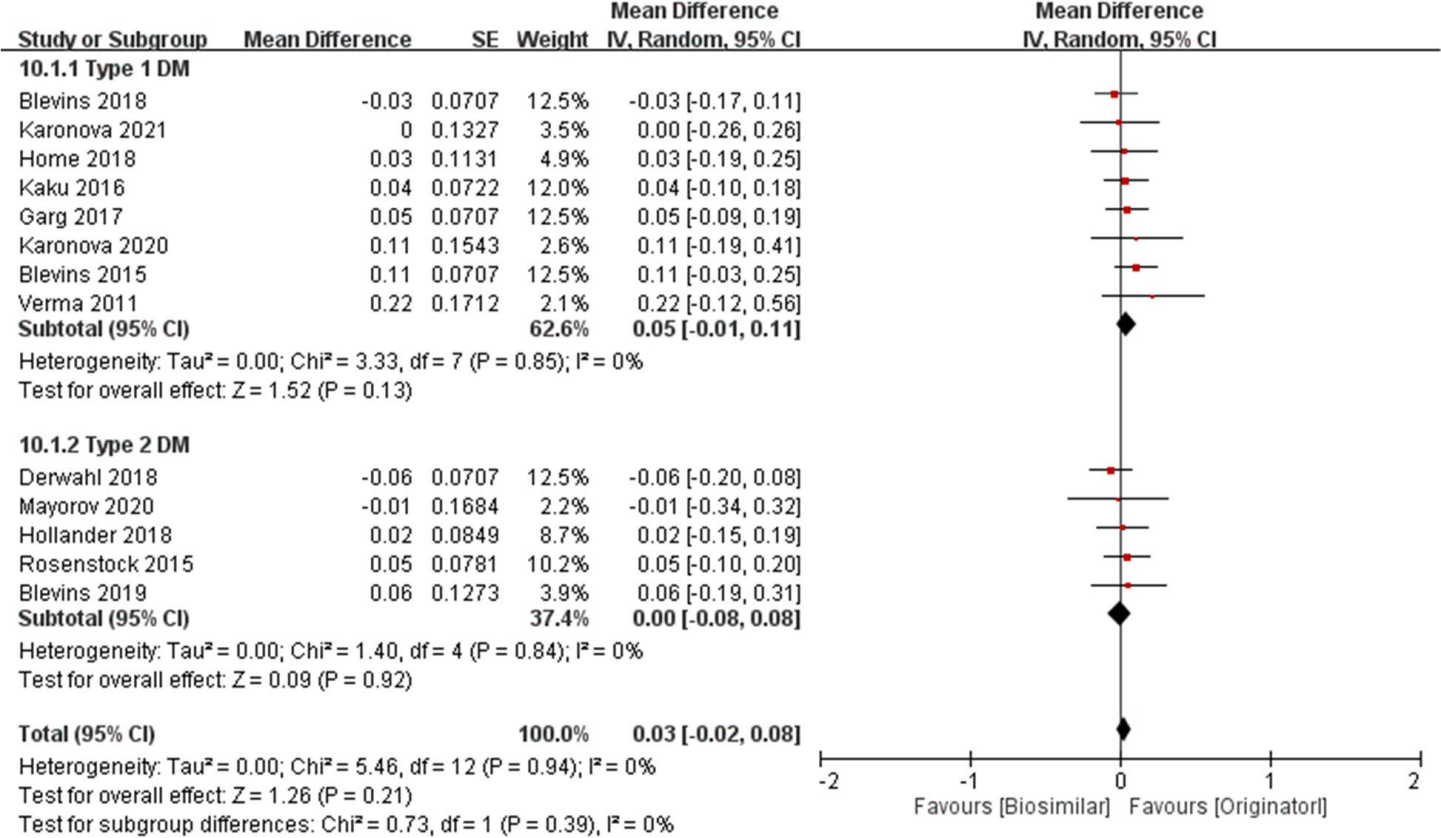
HbA1c at 26 weeks (%) in patients with type 1 diabetes and type 2 diabetes

Subgroup analysis was performed on duration of insulin and type of diabetes. The pooled MD of long- and short- acting insulin were 0.05 (95% CI − 0.01 to 0.11, *p* = 0.11) and − 0.01 (95% CI − 0.08 to 0.07, *p* = 0.80), respectively (Fig. 7). The pooled MD of type 1 DM and type 2 DM were 0.05 (95% CI − 0.01 to 0.11, *p* = 0.13) and 0.00 (95% CI − 0.08 to 0.08, *p* = 0.92), respectively (Fig. 8).

Fourteen studies were included in this study. A total of 11 included studies have adjusted with antidiabetic medications or the doses of insulin. Only 3 articles are not clear about whether there are adjusted with antidiabetic medications or the doses of insulin. Therefore, we will carry out a sensitivity analysis by excluding these 3 trials. When these 3 trials were removed from this meta-analysis, similar results were shown in a change of HbA1C at weeks 26 in comparison with the original one (MD: 0.03; 95% CI − 0.03 to 0.08, *p* = 0.33).

### Publication bias

**Fig. 9:**
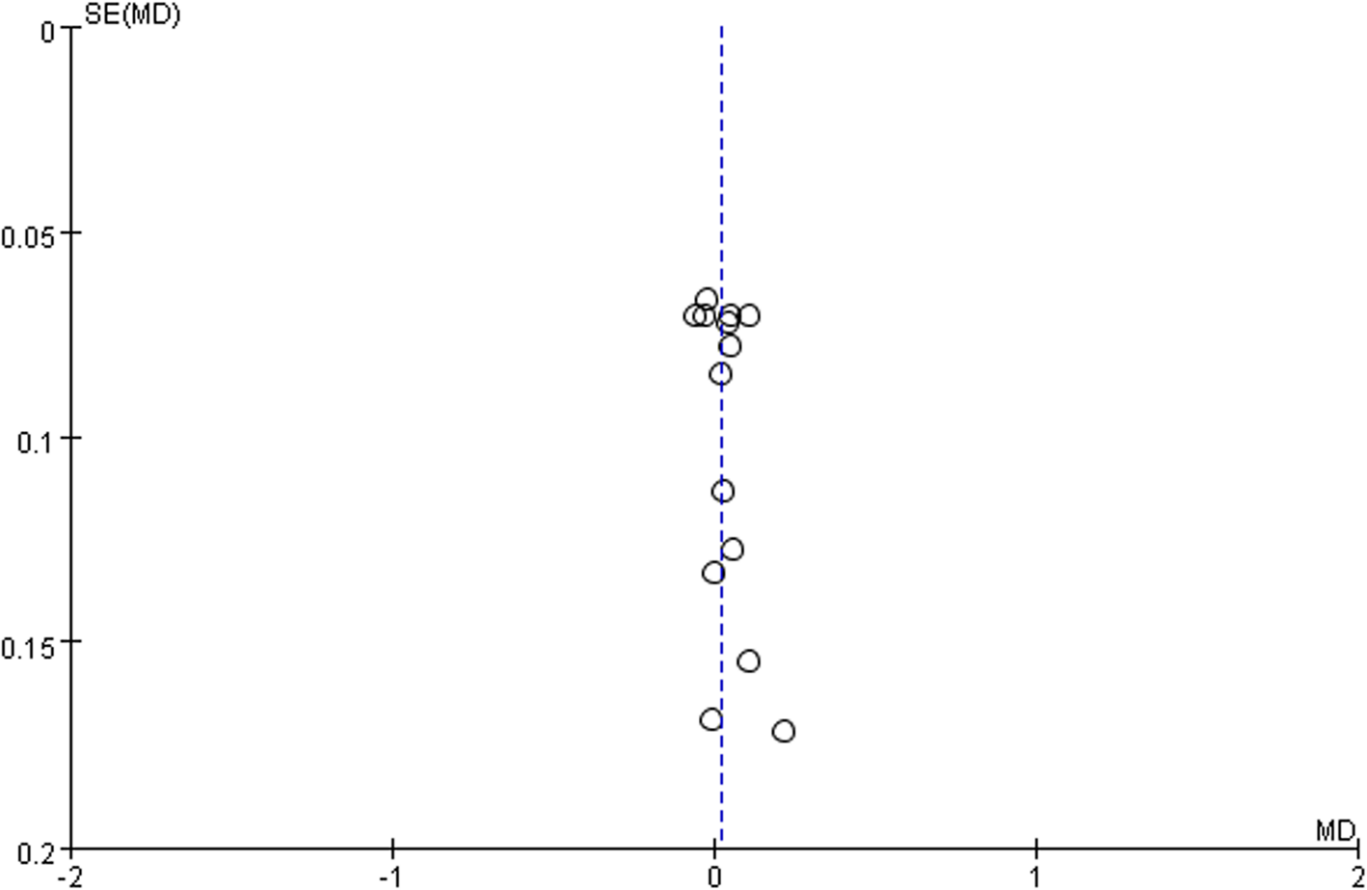
Publication bias funnel plot

A visual inspection of the funnel plot of results from these studies revealed asymmetry (Fig. 9). The results of Begg and Mazumdar rank (Kendall’s tau = 0.154 and *p* = 0.443) and Egger’s regression intercept approach (intercept = 0.752, two-tailed 95% CI: − 0.526 to 2.03, *p* = 0.224) indicated no significant evidence of publication bias in this meta-analysis.

## Discussion

In this current study, systemic review and meta-analysis were performed on 14 RCTs involving 6188 patients, studies that assess long- or short-acting insulin biosimilar with the originator, show no significant difference in effectiveness, safety, immunogenicity between the biosimilar and reference products. By contrast, previous observational studies show some biosimilar products (ex. granulocyte-colony stimulating factor) were associated with a higher rate of reportable adverse events and experienced drug ineffectiveness in post-marketing pharmacovigilance investigation [31]. Furthermore, some systemic review studies showed the Health Care Providers (HCPs) perspective in general of using biosimilar [32–34]. The knowledge and confidence of using biosimilar HCPs are varying between countries, health economics policies, clinical profiles, and studies. To prescribe biosimilar in initial treatment is still the most positive part for HCPs [32, 33]. The key concerns from HCP’s perspective whether to use biosimilar mainly focus on safety, efficacy, and immunogenicity. Furthermore, HCP’s knowledge gap regarding biosimilar itself, clinical outcome, harmonization of regulation and science, financial consideration…et al. have also influenced the prescription of biosimilars [32–34].

Previously, systemic review and meta-analysis estimated the efficacy of insulin biosimilar [35, 36]. A systemic review performed by Tai et al. includes 11 RCTs, which compared the safety, efficacy, pharmacokinetics (PK), and pharmacodynamics (PD) in both long-acting and short-acting between the biosimilar and their reference products. All parameters reported in PK and PD studies are within the acceptable margin and meet the requirements of similar [35]. Yamada et al. performed a systemic review and meta-analysis studies, which included 10 phases III RCTs and a total of 4935 participants and estimated long-acting biosimilar and their reference product [36]. The efficacy results of Yamada et al. and our study in change of HbA1C at 24–26 weeks, change of HbA1C at 52 weeks, and FPG decrease from baseline are 0.04 (95% CI − 0.01 to 0.08) versus 0.03 (95% CI − 0.02 to 0.07), 0.03 (95% CI − 0.04 to 0.1) versus 0.05 (95% CI − 0.05 to 0.15), and 0.08 (95% CI − 0.36 to 0.53) versus 0.02 (95% CI − 0.20 to 0.24), respectively. In addition, the safety results of Yamada et al. and our study in hypoglycemia and severe hypoglycemia are 0.99 (95% CI 0.96 to 1.03) versus 0.96 (95% CI 0.85 to 1.09) and 1.09 (95% CI 0.80 to 1.47) versus 1.06 (95% CI 0.85 to 1.31), respectively. This study includes newly RCTs [25, 28–30], pooled more comprehensive evidence in both long−/short acting insulin biologics, reports consistent outcomes than the previous study. However, our study grouped the studies with corrections combined with hypoglycemic drugs, and the results of the study were found to be similar (change of HbA1C at weeks 26, the MD were 0.03; 95% CI − 0.02 to 0.07, *p* = 0.28). The efficacy and safety are similar between insulin biosimilar and their originator. The price of insulin biosimilar is cheaper, it could benefit low-income countries to obtain medicines and use them.

There is some limitation to this study. First, as the administration of insulin is via Subcutaneous (SC), that leads to open-label design and might be unable to avoid investigator and participants bias of reports especially on safety, such as hypoglycemia and adverse events. Further unknown factors in the reported trials concerning trial quality and reduction of biases might have influenced the results of this meta-analysis. Second, different frequencies of tracking patients in outpatient visits of telephone follow-up could reflect inconsistency of medication adherence between trials. Third, we still did not report the issue of the interchangeable or switching issue in RCT based study.

## Conclusions

Insulin biosimilar showed comparable characteristics with the reference drug in terms of efficacy, safety, and immunogenicity through comprehensive and specific conventional meta-analysis, even in the subgroup analysis of the different types of diabetes and different duration of insulin. This systematic review and meta-analysis demonstrated the similarity of insulin biosimilar as a treatment for patients with both type 1 and type 2 diabetes. Our result can support the evidence-based use of insulin biosimilar and provide another positive choice on patient access to treatment.

## Supplementary Information

**Additional file 1: Appendix S1.** Searching strategy. **Figure S1.** Preferred Reporting Items for Systematic Reviews and Meta-Analyses (PRISMA) 2020 flow diagram. **Figure S2.** Methodological quality of the studies included in the final analysis based on Risk of Bias for assessing the quality of RCT (*n* = 14). **Table S1.** Clinical outcomes of the trials investigated.

## Data Availability

All data, models, and code generated or used during the study appear in the submitted article.
