# Supplementary material for: Efficacy and immunogenicity of insulin biosimilar compared to their reference products: a systematic review and meta-analysis
Source: BMC Endocr Disord. 2022 Feb 5;22:35. doi: 10.1186/s12902-022-00944-5 (PMC8817566; doi:10.1186/s12902-022-00944-5)
Supplement: Supplementary file 1 — Additional file 1: Appendix S1. Searching strategy. Figure S1. Preferred Reporting Items for Systematic Reviews and Meta-Analyses (PRISMA) 2020 flow diagram. Figure S2. Methodological quality of the studies included in the final analysis based on Risk of Bias for assessing the quality of RCT (n = 14). Table S1. Clinical outcomes of the trials investigated. [file 12902_2022_944_MOESM1_ESM.docx]

**Supplementary**

**Appendix S1.** Searching strategy

**Figure S1.** Preferred Reporting Items for Systematic Reviews and Meta-Analyses (PRISMA) 2020 flow diagram

**Figure S2.** Methodological quality of the studies included in the final analysis based on Risk of Bias for assessing the quality of RCT (n = 14)

**Table S1.** Clinical outcomes of the trials investigated

**Appendix S1 Search strategy**

**PubMed:**

(("biosimilar pharmaceuticals"[MeSH Terms] OR ("biosimilar"[All Fields] AND "pharmaceuticals"[All Fields]) OR "biosimilar pharmaceuticals"[All Fields] OR "biosimilar"[All Fields] OR "biosimilars"[All Fields] OR "biosimilarity"[All Fields]) AND ("insulin"[MeSH Terms] OR "insulin"[All Fields] OR "insulin s"[All Fields] OR "insuline"[All Fields] OR "insulinic"[All Fields] OR "insulinization"[All Fields] OR "insulinized"[All Fields] OR "insulins"[MeSH Terms] OR "insulins"[All Fields])) AND (randomizedcontrolledtrial[Filter])

**Cochrane library:**

(biosimilar) AND ("insulin") in Trials (Word variations have been searched)

**Embase:**

insulin AND biosimilar AND 'randomized controlled trial'


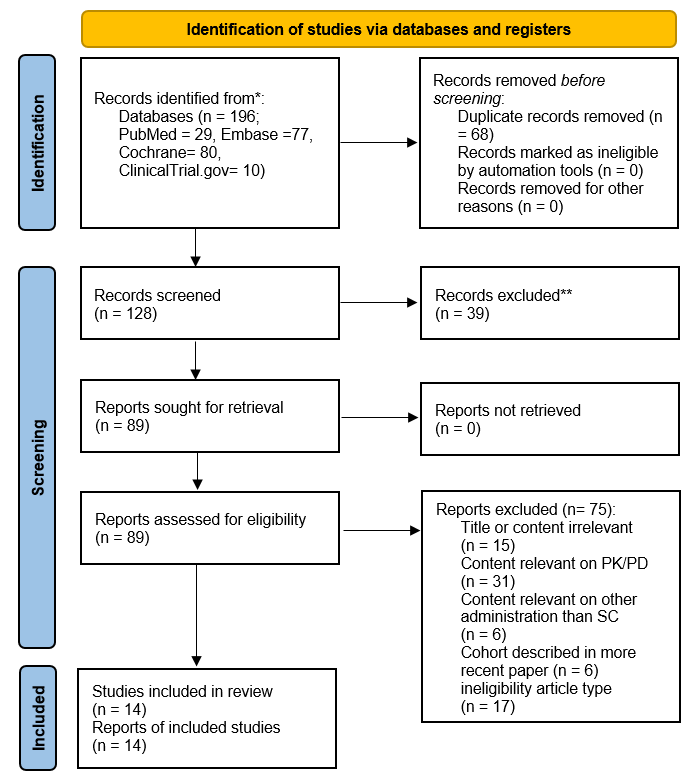


*Consider, if feasible to do so, reporting the number of records identified from each database or register searched (rather than the total number across all databases/registers).

**If automation tools were used, indicate how many records were excluded by a human and how many were excluded by automation tools.

**Figure S1.** Preferred Reporting Items for Systematic Reviews and Meta-Analyses (PRISMA) 2020 flow diagram.


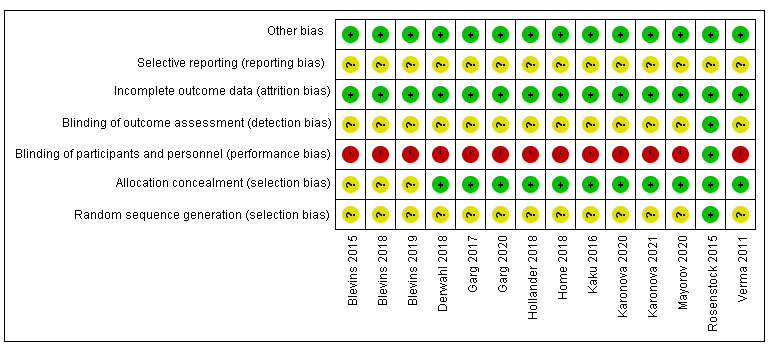


**Figure S2.** Methodological quality of the studies included in the final analysis based on Risk of Bias for assessing the quality of RCT (n = 14)

**Table S1.** Clinical outcomes of the trials investigated

| **Study, Year** | **Change of HbA1C** **(%)**  **24 -26 wks**  **MD (SE)** | **Change of HbA1C (%)**  **52 wks**  **MD (SE)** | **FPG (laboratory or SMBG)**  **24-52 wks**  **mmol/L**  **MD (SE)** | **SMBG (7-or 8- points)**  **24-52wks**  **MD (SE)mmol/L**  **MD (SE)** | **HbA1C < 7%**  **(d/N)** | | **hypoglycemia**  **(at least one event)**  **(%)** | | **Severe hypoglycemia (d/N)** | | **AIA_positive**  **(%)** | |
| --- | --- | --- | --- | --- | --- | --- | --- | --- | --- | --- | --- | --- |
|  |  |  |  |  | **BSM** | **REF** | **BSM** | **REF** | **BSM** | **REF** | **BSM** | **REF** |
| **Long-acting insulin biosimilar** | | | | | | | | | | | | |
| Verma, 2011 [17] | 0.22 (0.17) | NA | 1.03 (0.74) | NA | 34/107 | 36/108 | 43/107 | 45/108 | 3/107 | 3/107 | NA | NA |
| Kaku, 2016 [18] | 0.04 (0.07) | NA | 0.55 (0.40) | NA | NA | NA | 124/131 | 116/129 | 5/131 | 3/129 | NA | NA |
| Blevins, 2015 [19] | 0.11 (0.07) | NA | 0.17 (0.28) | NA | 81/268 | 67/267 | 230/268 | 235/267 | 11/268 | 11/267 | 107/268 | 105/267 |
| Rosenstock, 2015 [20] | 0.05 (0.08) | NA | -0.06 (0.24) | NA | 180/376 | 197/380 | 297/376 | 296/380 | 4/376 | 4/380 | 56/376 | 40/380 |
| Blevins, 2018 [21] | -0.03 (0.07) | 0.04 (0.08) | -0.9 (0.38) | 0.23 (0.14) | NA | NA | 154/280 | 170/278 | 11/280 | 13/278 | NA | NA |
| Home, 2018 [22] | 0.03 (0.11) | -0.02 (0.13) | 0.48 (0.65) | NA | 61/241 | 68/258 | 184/241 | 204/258 | 40/241 | 45/258 | 177/241 | 195/258 |
| Hollander, 2018 [23] | 0.02 (0.08) | NA | 0.19 (0.25) | NA | 112/241 | 107/245 | 140/263 | 137/263 | 4/263 | 4/263 | 91/262 | 76/262 |
| Blevins, 2019 [24] | 0.06 (0.13) | NA | 0.31 (0.26) | 0.35 (0.20) | NA | NA | 75/277 | 66/283 | 62/277 | 52/283 | 70/276 | 76/282 |
| Karonova, 2020 [25] | 0.11  (0.15) | NA | -1.3 (0.81) | NA | NA | NA | 72/90 | 73/90 | 4/90 | 9/90 | 26/90 | 27/90 |
| **Short-acting insulin biosimilar** | | | | | | | | | | | | |
| Garg, 2017 [26] | 0.05 (0.07) | 0.08 (0.08) | 0.05 (0.39) | -0.03 (0.20) | NA | NA | 250/253 | 254/254 | 34/253 | 34/254 | 100/253 | 109/254 |
| Derwahl, 2018 [27] | -0.06 (0.07) | NA | 0.05 (0.25) | -1.91 (0.19) | 107/253 | 102/252 | 173/253 | 188/252 | 6/253 | 4/252 | 68/253 | 66/252 |
| Garg, 2020 [28] | -0.02  (0.07) | NA | -0.32 (0.35) | NA | NA | NA | 291/301 | 285/296 | 12/301 | 10/296 | 95/296 | 104/292 |
| Karonova, 2021 [29] | 0 (0.13) | NA | -0.1 (0.62) | NA | 26/132 | 19/132 | 97/132 | 91//132 | 2/132 | 1/132 | 29/132 | 29/132 |
| **Pre-mixed insulin biosimilar** | | | | | | | | | | | | |
| Mayorov, 2020 [30] | -0.01 (0.17) | NA | -0.57 (0.58) | NA | NA | NA | 50/105 | 51/105 | 0/105 | 0/105 | 9/105 | 4/105 |
|  | | | | | | | | | | | | |
